# Polymer Flooding in Space-Constrained Reservoirs: Technical and Economic Assessment of Liquid vs. Powder Polymers

**DOI:** 10.3390/polym17212927

**Published:** 2025-10-31

**Authors:** Muhammad Tahir, Rafael E. Hincapie, Dominic Marx, Dominik Steineder, Amir Farzaneh, Torsten Clemens, Nikola Baric, Elham Ghodsi, Riyaz Kharrat

**Affiliations:** 1 OMV Exploration & Production GmbH, 1020 Vienna, Austria; 2 OMV Austria Exploration & Production GmbH, 2230 Gänserndorf, Austria; 3 Department Geoenergy (DGE), Montanuniversität Leoben, 8700 Leoben, Austriariyaz.kharrat@unileoben.ac.at (R.K.)

**Keywords:** liquid polymers, powder polymers, injectivity, emulsions, pilot project

## Abstract

This study evaluates the technical and economic feasibility of liquid polymer emulsions as substitutes for powder polymers in polymer flooding applications, particularly in space-constrained, low-permeability reservoirs in Austria. Rheological tests determined that target viscosities of 20 mPa·s at 20 °C and a shear rate of 7.94 s^−1^ were achieved using concentrations of 1200 ppm for liquid polymer 1 (LP1), 2250 ppm for liquid polymer 2 (LP2), and 1200–1400 ppm for powder polymers. Injectivity tests revealed that liquid polymers encountered challenges in 60 mD and 300 mD core plugs, with pressure stabilization not achieved at injection rates of 1–2.5 ft/day. Powder polymers demonstrated stable injectivity, with powder polymer 1 (PP1) showing an optimal performance at 10 ft/day and a low residual resistance factor (RRF). Two-phase core floods using PP1 and powder polymer 2 (PP2) at 1 ft/day yielded incremental oil recovery factors of approximately 5%, with a maximum of 8% observed for higher viscosity slugs. Economic analysis indicated that over a 3-year horizon, liquid polymers are 30% cheaper than powder polymer Option 1 but 100% more expensive than Option 2. Over a 10-year horizon, liquid polymers are 50% more expensive than both powder polymer options. Although liquid polymers offer logistical advantages, they are unsuitable for low-permeability reservoirs. Powdered polymers, particularly PP1, are recommended for pilot implementation due to superior injectivity, mechanical stability, and recovery performance.

## 1. Introduction

Polymer flooding has become popular as a chemical enhanced oil recovery (CEOR) approach [1] in recent years. Adding polymer to brine increases the viscosity of the water phase and improves displacement efficiency by reducing the mobility ratio. Thus, the synergistic impact of a reduced mobility ratio and enhanced sweep efficiency facilitates oil recovery compared to conventional water flooding [2,3,4]. Increased water viscosity improves microscopic, vertical, and areal sweep efficiency by removing issues such as fingering and early water breakthrough [5,6].

### 1.1. Polymer Types

The petroleum industry primarily uses two types of polymers: synthetic polymers (such as hydrolyzed polyacrylamide, or HPAM) and biopolymers (like xanthan gum). Synthetic polymers are manufactured in an industrial setting to meet specific industrial requirements, whereas natural microbial fermentation processes produce biopolymers.

Biopolymers gained interest in the 1970s and 1980s because of their improved tolerance to mixing brine hardness and resilience to mechanical degradation [7,8]. However, their field-scale deployment has been limited due to their high cost, limited commercial-scale supply, and consumption by reservoir microbes [9,10,11,12,13,14]. Synthetic polymers, on the other hand, are now the product of choice for industrial applications because of their viscoelasticity, increased viscosity, low cost, and strong resistance to microbial activity. The most commonly used synthetic polymer in EOR projects is partially hydrolyzed polyacrylamide (HPAM). Several research projects have developed HPAM-based terpolymers and copolymers to overcome HPAM’s susceptibility to salinity, brine hardness (divalent cation), and reservoir temperature [15,16,17,18].

### 1.2. Challenges in Low-Permeability Reservoirs

Researchers have investigated the use of modified HPAM-based synthetic polymers in challenging reservoir conditions [19,20,21]. However, traditional powder polymers require a two-step hydration process to produce the mother solution and the diluted solution; their field-scale deployment requires significant space for the mixing unit. The use of powder polymer flooding, therefore, has limitations for offshore applications and small onshore reservoirs (which require larger capital expenditures) due to these space constraints.

Polymer Alternating Water (PAW) is a hybrid injection technique used in chemical enhanced oil recovery (CEOR). Instead of continuously injecting polymer, PAW alternates between polymer slugs and water slugs, aiming to optimize oil displacement while minimizing polymer consumption. By alternating, PAW can potentially reduce overall costs and improve sweep efficiency by using water to push oil, while the polymer helps to improve the mobility ratio and sweep more of the reservoir. Recent studies suggest that contemporary methods, such as PAW, can be utilized to optimize polymer flooding in low-permeability reservoirs. This technique lowers polymer consumption and increases sweep efficiency by injecting water slugs between polymer slugs. PAW technology has been demonstrated to enhance oil recovery and improve control over polymer adsorption in low-permeability zones, making it an effective approach for addressing challenging reservoir conditions [5]. Moreover, low-molecular-weight amphiphilic polymers have been developed to solve injectivity problems in low-permeability reservoirs. These polymers significantly enhance oil recovery through their improved thickening capacity, shear-thinning behavior, and increased oil–water interfacial activity [6].

### 1.3. Liquid Polymers and Field Constraints

Liquid polymers (LPs), also known as emulsion polymers, represent a novel class of materials synthesized via water-in-oil emulsions. They are distinguished by their ease of handling, rapid deployment, and compact surface facilities, making them particularly suitable where powdered polymers may be impractical due to space constraints or high initial investment costs [22]. Liquid polymers are synthesized through a water-in-oil emulsion method, employing any commercially available powdered polymer as a viscosity-enhancing base product [23]. Inversion surfactants are vital in LP formulations since they enable emulsion inversion upon mixing with brine at higher shear rates; hence, they release the polymer for hydration and enhance the viscosity of the aqueous phase. LPs comprise a powdered polymer coated in an emulsion phase, which consists of mineral oil and surfactant, and is dispersed in a liquid medium. Consequently, a one-step hydration procedure (static mixer) is necessary to prepare a diluted solution. The benefit of these static mixers is their small space requirement and their ability to generate dilute solutions of desired concentration by directly utilizing 50% of the active polymer product.

Nevertheless, additional chemicals are required to maintain LP stability and prevent phase separation, emulsion degradation, and microbial growth [24]. pH modifiers are incorporated into the LP as needed to make the solution either alkaline or acidic [25]. The combination of various chemicals leads to a higher cost per unit of the final LP product than powdered polymers. However, powdered polymers require higher capital investments for plant installation and operation [26]. Moreover, the presence of certain chemicals in the diluted LP solution might limit injectivity or lead to excessively high residual resistance factors (RRF) [22,27]. Significant efforts have been made to address injection challenges through the development of modified liquid polymers for the Captain field in the North Sea. In their core flood tests, Osterloh and Law [27] utilized sand packs with an average reservoir permeability of 7000 mD, and Dwarakanath et al. [22] employed Bentheimer core plugs with higher permeabilities ranging from 1300 to 2000 mD. Consequently, it remains uncertain whether the recommended LPs reported by these two studies are suitable for injection into low-permeability reservoirs (50–300 mD).

### 1.4. Study Rationale and Objectives

Several small oil fields in Austria have lower permeabilities compared to the Captain’s field. Previous studies on liquid polymers (LPs) have primarily focused on high-permeability reservoirs, leaving uncertainty about their applicability in low-permeability conditions. This study addresses that gap by evaluating the technical and economic feasibility of LPs versus powder polymers in an Austrian reservoir with an average permeability of 550 mD. Given the small size and space constraints of the field, an LP flooding trial is proposed to minimize capital expenditure using a containerized unit. If successful, similar trials will be extended to other small fields.

To ensure a comprehensive comparison, the powdered polymer—used as the base for LP formulation—will also be tested under identical conditions. The study aims to determine the optimal polymer type for pilot implementation in space-constrained reservoirs by assessing injectivity, performance, and cost-effectiveness. This dual evaluation will help identify the most suitable polymer flooding strategy for low-permeability Austrian oil fields.

## 2. Approach and Methods

We employed a multi-step experimental approach to evaluate the performance of liquid and powdered polymers in enhanced oil recovery (EOR), focusing on both rock–fluid and fluid–fluid interactions. The methodology was designed to simulate reservoir conditions and assess key parameters influencing polymer selection:

- Rheological Characterization: The polymers’ rheological properties are characterized through concentration and temperature measurements.
- Fluid–fluid Interaction: Interfacial tension measurements and the emulsion stability assessment are performed on the liquid polymers to analyze the impact of surfactants.
- Injectivity Screening: Single-phase core flood tests are performed to assess the injectivity of the liquid and powdered polymers.
- Two-phase Core flood: Employing the selected polymers from the injection screening, experiments are performed at lower rates to evaluate recovery and injectivity.
- Decision Matrix Development: A comparative decision matrix was constructed based on experimental outcomes, including injectivity, mechanical stability, and recovery efficiency, to support polymer selection for pilot implementation.

## 3. Reservoir Data and Materials

### 3.1. Rock Properties

This feasibility study for polymer flooding targets the 11 Tortonian Horizon (TH) Sarmatian reservoir in the Hochleiten field. The reservoir comprises alluvial to fractured channel sandstones, shaped by its sedimentary environment. Water injection is used to maintain reservoir pressure, achieving a recovery factor between 33% and 49%. Initial pressure was 82 bar, now reduced to 25 bar. Reservoir temperature at the production well was 36 °C, dropping to 25 °C at injection wells due to water injection.

Core plug permeability ranges from 20 mD to 4000 mD, averaging 550 mD. Tracer tests in 2020 confirmed connectivity between injection and production wells. Given the reservoir’s location in an environmentally sensitive area with limited space for surface facilities, liquid polymer injection is a suitable option to minimize footprint.

Due to limited availability of actual core plugs and the presence of unconsolidated rock, single-phase core flood experiments were conducted using three types of sandstone outcrop plugs to simulate reservoir heterogeneity. Berea sandstone plugs with brine permeabilities of 60 and 300 mD represent low-permeability zones, while Liver Gray plugs with 550 mD reflect average reservoir permeability. These plugs measured approximately 7 cm in length and 3.8 cm in diameter.

For two-phase coreflood experiments, longer Berea sandstone plugs (30 cm length, 3.8 cm diameter) were used to overcome the capillary end effects and dead volume discrepancies. The 300 mD Berea core plugs are chosen for the two-phase corefloods based on mercury intrusion capillary pressure (MICP) and contact angle assessments. Furthermore, two actual reservoir cores (2 cm in length and 3 cm in diameter) are utilized to compare outcrop cores, one representing the low permeability region of the target reservoir and the other reflecting the average permeability of the target reservoir.

### 3.2. Fluid Properties

Brine from the 8 TH reservoir coming from Water Treatment Plant (WTP) is utilized for core flooding and polymer slug preparation. The brine has a viscosity of 0.997 mPa·s at 25 °C. Table 1 describes the chemical composition of the utilized brine. The reservoir’s formation brine is soft and has a low total dissolved solids (TDS) content, and 8 TH WTP water injection activity at the injector well commenced in 2019. It is anticipated that in the majority of the flooded area of the target reservoir, the formation brine is either mixed with or displaced by the injected brine.

Crude oil from well HL 20 is combined with 1.55 wt% cyclohexane to achieve a viscosity of around 80 mPa·s, needed for two-phase coreflood experiments under present reservoir conditions. Crude oil is used for fluid–fluid interaction and contact angle measurements. The crude oil exhibits reactivity, possessing a total acid number (TAN) of 1.56 mg KOH/g and an API gravity of 19.97 (°). The SARA analysis indicated 36.2% saturates, 53.2% aromatics, 9.2% resins, and 1.4% asphaltene.

Polymer slugs are produced using commercial polymer products from two vendors: SNF and Sterling Specialty Chemicals (ex-Kemira). However, due to chemical confidentiality, the vendors are referred to as A and B, and the commercial product names and formulas are not disclosed. The research initially focused on liquid polymers, with each supplier recommending an emulsion polymer product specifically tailored to the reservoir conditions. Due to the injection difficulties associated with liquid polymers, four commercial powder polymer products (two from each vendor) are subsequently incorporated into the feasibility study. Table 2 illustrates that the evaluation process focused on the following polymer products, which are suggested by vendors and within the company. Concentrations of polymer products are adjusted to attain a final target viscosity of 20 mPa·s at 20 °C and a shear rate of 7.94 s^−1^.

## 4. Methods

### 4.1. Polymer Slug Preparation

#### 4.1.1. Liquid-Polymer Solutions

We performed a two-step hydration technique to prepare the diluted LP solution, as advised by both vendors. On-site hydration of these polymers is a one-step process, whereas the laboratory preparation approach requires a two-step procedure in order to achieve a homogeneous solution. Initially, a more concentrated stock solution is produced. The active polymers must be agitated and blended extensively, as the primary active polymer is heterogeneous, and the solid phase will precipitate after more than a day of storage. The provider specified an emulsion concentration of 10,000 ppm for LP#1 and 20,000 ppm for LP#2 for the stock solution. The necessary polymer weight to prepare the stock solution is determined using the following formula:

(1) $$Needed\;polymer\;amount\left(g\right)=\frac{Target\;concentration\left(ppm\right)\times Total\;weight\left(g\right)}{Active\;polymer\;concentration\left(ppm\right)}$$

The manufacturer specifies active polymer concentrations of 500,000 ppm for LP#1 and 1,000,000 ppm for LP#2. The desired concentrations for stock solutions are 10,000 ppm and 20,000 ppm, respectively. Upon measuring the active polymer and the required weight of 8 TH WTP brine, the solutions are stirred at 500 rpm for 2 h. The stock solutions are diluted to the desired concentrations using the following formula:

(2) $$Needed\;mother\;solution\;amount\;\left(g\right)=\frac{Total\;weight\left(g\right)\;\times \;Target\;concentration\left(ppm\right)}{Mother\;solution\;concentration\;\left(ppm\right)}$$

#### 4.1.2. Powder-Polymer Solutions

Likewise to LP, powder polymer solutions have been produced by applying a two-step hydration process as described in previous studies [28,29]. Initially, a 5000 ppm stock solution was prepared. Diluted solutions at the required concentrations were prepared using the same methodology as for LP.

#### 4.1.3. Rheological Measurements:

Viscosity was measured using a Kinexus Pro+ concentric cylinder rheometer. A concentration scan established the requisite polymer concentration to attain a slug viscosity of 20 mPa·s at 20 °C and a shear rate of 7.94 s^−1^. Considering the low temperature at the injection well (25 °C) and the high temperature deep in the reservoir (36 °C), a temperature scan was conducted from 20 °C to 40 °C to examine how polymer viscosity reduces with increasing temperature. Fresh samples were used for each measurement, and the viscosity was measured at different shear rates from 1 s^−1^ to 20 s^−1^. The viscosity was measured at a shear rate of 7.94 s^−1^ at a specific concentration or temperature, providing a comparison of polymer products.

### 4.2. Fluid–Fluid Interactions

#### 4.2.1. Interfacial Tension Measurements (IFT)

Measurements of interfacial tension (IFT) are required to look at potential chemical reactions between LP and crude oil since LP composition includes small amounts of surfactants and emulsions, whereas crude oil contains reactive compounds. LP IFT measurements were conducted using a spinning drop tensiometer. Oil samples from an 11 TH Sarmatian reservoir have been added to an in-house prepared polymer solution. To reduce measurement error, three trials were conducted with each solution. Mean values and standard deviation were calculated. Repeated measurements were performed using LP-free brine from 8 TH reservoir as the benchmark.

#### 4.2.2. Phase Behavior Evaluations

Phase behavior studies were conducted on the two LPs to further examine fluid–fluid interactions. We employed the methodology described elsewhere [30]. Samples for phase behavior tests were prepared in 10 mL pipettes and sealed using a methane-oxygen flame. The pipettes were filled with diluted LP and the crude oil and subsequently flame-sealed from the top. The pipettes were rotated at 50 rpm for 48 h on a rotary shaker to guarantee comprehensive mixing. The samples were stored in an oven at 36 °C, and the volumes of the various phases in each sample were observed. To improve the data reliability, multiple tests were conducted for each solution.

#### 4.2.3. Liquid-Polymer Stability Tests

The bottle test is carried out at 36 °C to assess polymer stability for an extended duration. Unstable solutions may result in phase separation and flocculation. The polymer viscosity is measured over days while the liquid bottle is kept at 36 °C to confirm thermal stability. Thermal stability (degradation) is determined using the following formula:

(3) $$Thermal\;Degradation\;Rate\left(\%\right)=\left(\frac{\eta_{Fresh\;solution}-\eta_{Sample\;from\;the\;oven}}{\eta_{Fresh\;solution}}\right)\times 100$$

where η represents the shear viscosity of the solution at a shear rate of 7.94 s^−1^ and 36 °C.

### 4.3. Rock–Fluid Interactions

#### 4.3.1. Single-Phase Core Flooding

**Figure 1:**
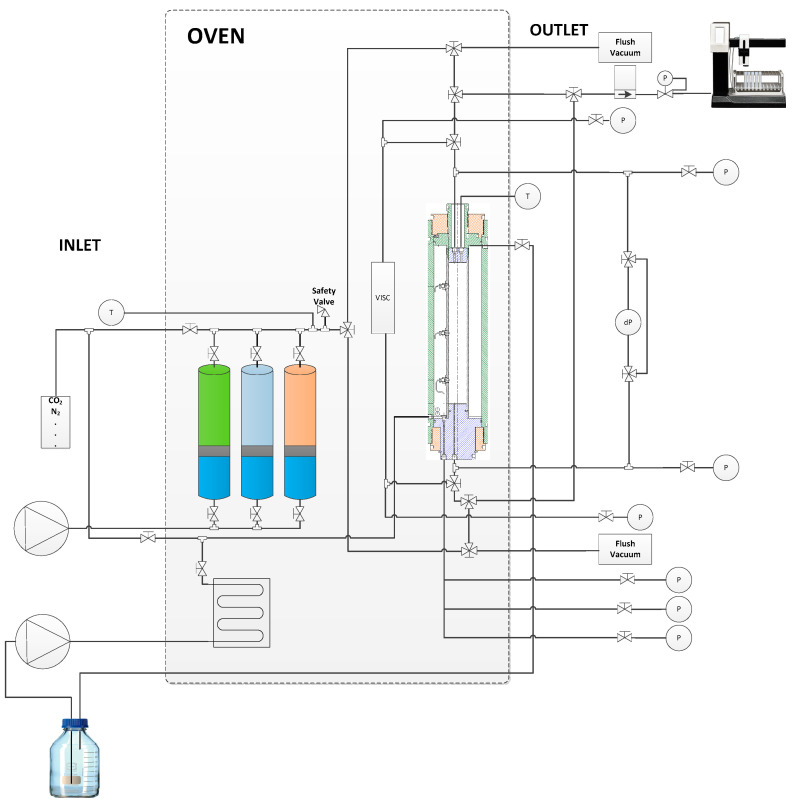
Experimental setup used for core flooding experiments.

Injectivity Test: Single-phase coreflood tests were performed utilizing a custom-designed apparatus, as shown in Figure 1. The cores were initialized using routine core analysis methodology. The cores were placed in a holder and subjected to a radial confining pressure of 40 bar(g) and a pore pressure of 5 bar(g). Carbon dioxide injection continued for 30 min. The cores were saturated with synthetic formation brine at a pore pressure of 5 bar(g). The oven temperature was gradually increased to 25 °C (recorded at the injection well), and brine permeability was determined. The sample was subsequently unloaded and weighed, and the pore volume was determined via the Archimedes method.

The injection test comprised saturating the core at 25 °C (the temperature of the injection well), introducing brine into the core, and measuring the pressure response. A stepwise injection rate was employed to measure the brine permeability of the core. During the second phase, a chemical (polymer) slug was injected at a rate of 5 ft/day or 10 ft/day for 24 h. The injection rate was modified to collect effluents, assess mechanical degradation, and examine the impact of viscoelasticity. During the last stage of the single-phase coreflood experiment, synthetic formation brine was reinjected. Table 3 describes the polymers and core plugs utilized in injection screening.

Resistance factor (RF) and residual resistance factor (RRF) were calculated to quantify the flow restriction imposed by aqueous phase adsorption on the rock. The formulas shown below were used to determine these two parameters:

(4) $$RF=\frac{{\Delta Ρ}_{POLYMER}}{{\Delta Ρ}_{BRINE\_PRE}}$$

(5) $$RRF=\frac{{\Delta Ρ}_{BRINE\_POST}}{{\Delta Ρ}_{BRINE\_PRE}}$$

where

ΔΡ POLYMER is the pressure drop for polymer flood.
ΔΡ BRINE_PRE is the pressure drop for brine flood before polymer injection at the similar injection rate.
ΔΡ BRINE_POST is the pressure drop for the brine flood after polymer injection at a similar injection rate.

For a single-phase core flood, polymer injection with better injectivity is expected to exhibit a stable differential pressure after breakthrough at a specific injection rate. Once this stable pressure is achieved, the value is used to calculate the resistance factor (RF), an indicator of injectivity. However, unstable differential pressure suggests an injectivity issue—for example, a constant increase in pressure without reaching a stable value may indicate filtration problems, improper dissolution, or plugging of the porous media. Similarly, a decline in differential pressure is expected during the post-brine flood, as the brine displaces the polymer from the porous media. Once a stable differential pressure is achieved after injecting significant pore volumes of post-brine, the Residual Resistance Factor (RRF) is calculated using Equation (5). However, no decline or increase in differential pressure during the post-brine stage is unexpected and may indicate injectivity issues.

Mechanical Degradation of Polymer Slug: Polymers experience mechanical degradation while traveling through porous media, leading to viscosity reduction. Comprehending the correlation between mechanical degradation and injection rate is essential, particularly in the vicinity of injection wells and under deep reservoir conditions. Consequently, assessing mechanical degradation is the primary objective of this research. The rate of mechanical degradation of solutions is calculated using the following formula:

(6) $$Mechanical\;Degradation\;Rate\left(\%\right)=\left(\frac{\eta_{Fresh\;solution}-\eta_{Effluent\;from\;core}}{\eta_{Fresh\;solution}}\right)\times 100$$

where η represents the shear viscosity of the solution at a shear rate of 7.94 s^−1^ and 36 °C.

To mimic the mechanical degradation caused by high injection rates in the vicinity of the injection well, polymer injection at high rates (10–15 ft/day) was accomplished in the core plug. Lower injection rates (1–5 ft/day) were employed to replicate deep-in-reservoir conditions. The rheological characteristics of the polymer solutions were assessed at 25 °C with a Kinexus Pro+ rheometer. Viscosity measurements were acquired at shear rates between 1 s^−1^ and 20 s^−1^.

#### 4.3.2. Two-Phase Core Flooding

Initialisation of Core Plugs: Conforming with the single-phase core flood procedure, a brine saturation phase and routine core analysis were conducted. For the two-phase core flood, a longer core plug (1 foot in length, 3.8 cm in diameter) was chosen to mitigate capillary end effects and minimize potential dead volume errors. Following measurement of brine permeability, the sample was saturated with crude oil until an initial brine saturation of approximately 30% was achieved. Differential pressure was closely monitored to detect any blockage caused by undesired particles in the injected oil. Following fluid initialization (oil/brine), effective oil permeability was assessed.

The Temporal Sequences: All slugs were injected at 1 ft/day interstitial velocity. The injection sequence format was as follows:

1. Injection of synthetic formation brine (8 TH WTP) at a volume not exceeding two pore volumes.
2. Injection of chemical (polymer) slugs for two pore volumes.
3. The post-brine (8th WTP) flood lasted for approximately 5 pore volumes.

Upon completion of the flooding experiment, core samples were retrieved. The produced effluents were collected in approximately 5 mL fraction tubes, and phase volumes were determined through visual inspection of the liquid level. This approach was utilized for both the oleic and aqueous phases in the graduated fractionator. The time and pressure differentials for each experiment were recorded. Table 4 outlines the general experimental parameters and fluids employed.

### 4.4. Cost Comparison and Logistical Evaluation of Polymer Selection

Liquid polymer (LP) has been chosen as the preferred cEOR technology owing to space constraints, the small size of the target reservoir, and lower capital expenditures. Nevertheless, due to the prior lack of LP application in low-permeability reservoirs, powdered polymers were also evaluated for testing and comparison. An economical assessment of the various possibilities for both polymer types was completed prior to technology qualification. Three economic scenarios were developed based on three potential outcomes.

#### 4.4.1. Scenario 1—Liquid Polymers

This approach employs a one-step hydration process to produce a diluted solution. The unit comprises two vessels measuring 20 × 8 ft (about 6 m × 2.4 m) each, with a maximum height of 10 ft (nearly 3 m). One vessel comprises a 24-cubic-meter ISO storage tank, whereas the other has all the mixing components, including a high-pressure pump and a tank for appropriate dilution with injection water. An ISO tank with a capacity of 24 cubic meters carries a highly concentrated solution (50% of the active polymer product) for LP. A static mixer dilutes the solution to the desired viscosity, while a small pump injects the target solution. A major constraint of this product is its feasibility for injection into low-permeability reservoirs. Moreover, the unit price (€/kg) of the LP product is roughly 60% pricier than that of the powdered polymer, and since the active component concentration in the LP product is roughly 50% of that in the powdered polymer, a larger quantity of LP is required. Nevertheless, this technology reduces the initial capital expenditure of the polymer facility. Additionally, the unit can be placed directly at the wellhead, minimizing the risk of polymer solution degradation and residue migration along the pipeline wall. Investment expenditures can be reduced if pipelines are unnecessary. The system is more portable owing to its compact footprint and containerized design.

#### 4.4.2. Scenario 2—Powder Polymer Option 1

The procedure involves a two-stage hydration procedure utilizing two 40 × 8-foot storage tanks. Powdered polymer is combined with mixing brine to create a high-viscosity mother solution, which is completed in tanks until completely matured. This solution is subsequently combined in a high-pressure brine stream to achieve the desired viscosity. The unit needs more room because of the massive tanks and the large number of personnel. The primary obstacle is the significant capital expenditure required for the polymer plant, despite polymers’ reduced operational costs. Consequently, owing to significant footprint and space limitations, the plant cannot be situated immediately at the wellhead. It must instead be situated close by, and a 1.5 km pipeline must carry the solution to the injection well.

#### 4.4.3. Scenario 3—Powder Polymer Option 2

This approach requires a more complex logistic strategy. High-concentration mother solution is brought by truck to a designated location in the target region (it cannot be positioned directly at the wellhead) and stored in a heated tank with a capacity of 100 cubic meters. The mother solution is combined with brine, diluted to the desired viscosity, and conveyed through a 1.5 km pipeline. A considerable risk in this scenario is the quality of the mother solution delivered to the wellsite. The possibility of oxygen exposure during loading and unloading might compromise the solution quality. Moreover, dangerous compounds present in transport tanks and insufficient cleaning may risk injection efficiency.

#### 4.4.4. Scenario Comparison

Outcomes can differ considerably based on project duration.

- Three-year period: After three years, Option 2 (the powder polymer plant) costs the most, almost 30% more than Option 1 (LP) and nearly 100% more than Option 3.
- Decade analysis: Over a decade, Options 2 and 3 expenses align; however, Option 1 costs approximately 50% more than the other two alternatives.

The decision depends on the project’s duration and additional criteria, including the space required for the plant and operator availability. Additionally, the expenses and contamination linked to several weekly transportation journeys must be considered. Constant trucking costs are utilized for simplicity, without adjustment for inflation. Polymer consumption is estimated at 120 kg per day for Options 2 and 3 and 240 kg per day for Option 1, based on 50% active polymer content. Pipeline expenses are not taken into account because pipes are already existing, which lowers the total initial expenditure needed.

## 5. Experimental Performance Results

### 5.1. Rheological Measurements

A concentration scan was performed to determine the target polymer concentrations that would lead to a slug viscosity of 20 mPa·s at 20 °C and a shear rate of 7.94 s^−1^. The analysis indicated the required concentrations of 1200 ppm for LP1, 2250 ppm for LP2, 1400 ppm for PP1, 1200 ppm for PP2, 1300 ppm for PP3, and 1400 ppm for PP4, as shown in Table 3. The concentration scan indicates that PP2, with the highest molecular weight (20–22 million Dalton) among the powder polymers, needs the lowest concentration to attain the desired viscosity. In contrast, PP1 and PP4, being the lowest molecular weight (18–20 million Dalton), require a higher concentration to reach the desired viscosity. Establishing a correlation between the molecular weights of the LP products is challenging due to suppliers utilizing differing calculation methodologies based on the stated active product concentration. The vendor states that LP1 utilizes PP1 as the basis for emulsion preparation, whereas LP2 uses PP2 as the base polymer. Consequently, the molecular weight of LP2, derived from the base powder polymer, is slightly higher than that of LP1.

**Figure 2:**
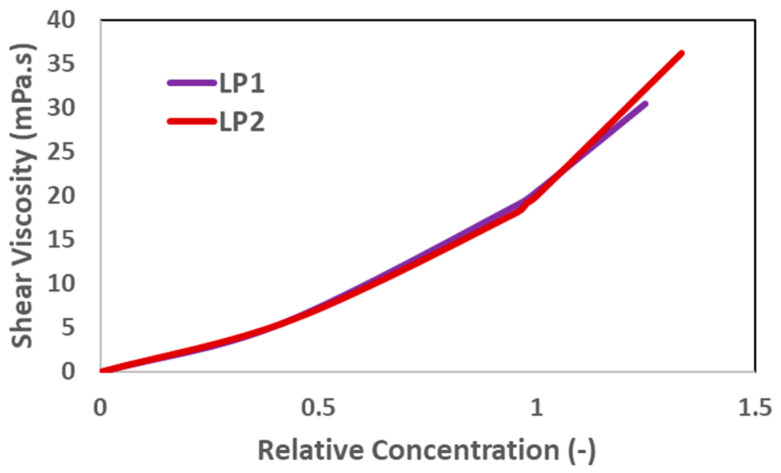
Relative concentration scan for liquid polymers. Shear viscosity is measured at a shear rate of 7.94 s^−1^ and at 25 °C.

Figure 2 illustrates the shear viscosity of liquid polymers in relation to relative concentrations. Relative concentration is defined as the specific concentration divided by the target concentration, which is 1200 ppm for LP1 and 2250 ppm for LP2. The two liquid products demonstrate comparable characteristics, as their curves are consistent and display similar patterns. Moreover, implementing the same concentration calculation method to get the desired viscosity, the use of LP2 is 2 wt% less than that of LP1, aligning with the molecular weight information of the powdered polymers.

**Figure 3:**
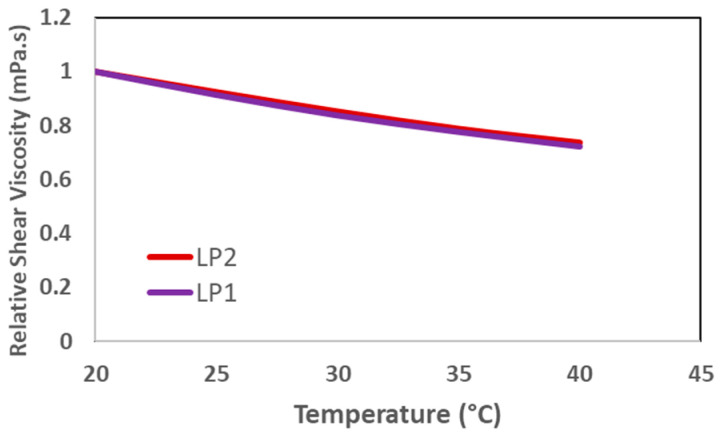
Temperature scan for liquid polymers. Shear viscosity measured at a shear rate of 7.94 s^−1^.

As predicted, Figure 3 illustrates a reduction in polymer viscosity due to increasing temperatures. The viscosity of the polymer slugs for both LP products declined by an equivalent degree. Moreover, it has been proven that the polymer viscosity under deep reservoir conditions will be less than 20 mPa·s owing to the elevated reservoir temperature. The relative shear viscosity depicted in Figure 3 is defined as the viscosity at a specific temperature divided by the viscosity of the polymer slug at 20 °C. Table 5 summarizes temperature scans for all polymer products. All polymer products demonstrated comparable viscosities at a specific temperature. The target viscosity was established with 20 °C as the reference, 25 °C as the injection well temperature, and 36 °C as the temperature deep in the reservoir.

### 5.2. Fluid–Fluid Interactions

**Figure 4:**
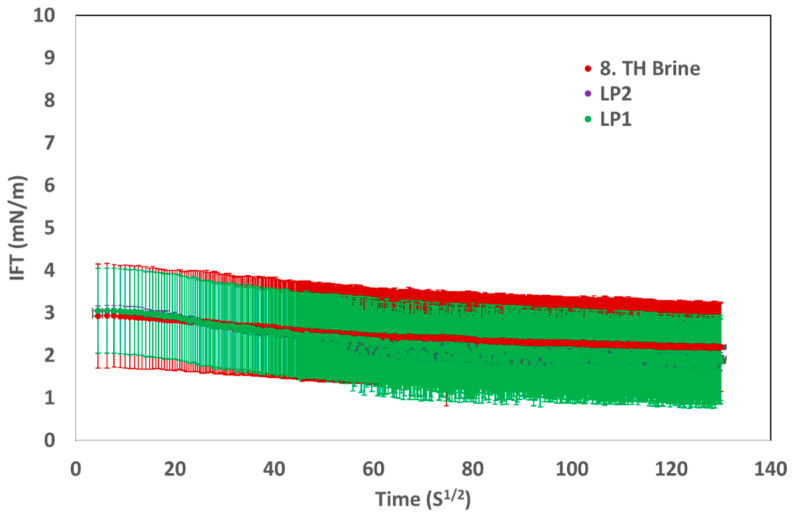
IFT measurements performed for both liquid polymers and for the 8 TH WTP brine (as the base case).

For fluid–fluid interactions, 1200 ppm LP1 and 2250 ppm LP2 were used, as these concentrations achieved a target viscosity of 20 mPa·s at 20 °C and a shear rate of 7.94 s^−1^. Figure 4 illustrates the interfacial tension (IFT) results for the two liquid polymers. Baseline measurements were carried out using 8 TH WTP brine for IFT assessments.

**Figure 5:**
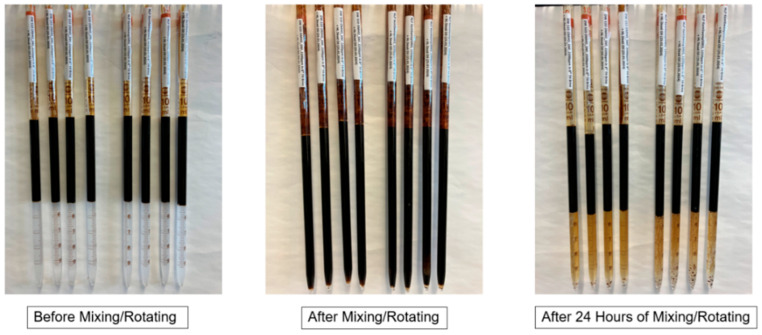
Phase behavior test using pipettes to produce the emulsions as the result of fluid–fluid interaction. In each picture the four pipettes on the left side are for the 2250 ppm LP2 and the four pipettes on the right side are for the 1200 ppm LP1.

Figure 4 illustrates that there is no significant difference in interfacial tension (IFT) measurements between the baseline solution and the liquid polymer. Consequently, it can be assumed that, at the specified concentration, the surfactant in the liquid polymer fails to interact with the crude oil and does not facilitate fluid–fluid interaction. Moreover, Figure 5 illustrates that no emulsion is produced in the pipette during the rotation of the liquid at a 1:1 volume ratio. After a few hours, a distinct separation of the oil and water phases happens. Consequently, it can be inferred that the interfacial tension (IFT) measurements and phase behavior analysis exclude evidence of fluid–fluid interaction.

Due to the inert nature of powdered polymers for crude oil and the lack of interfacial reactions, fluid–fluid interactions with the powdered polymers were not investigated [31,32]. IFT measurements and phase behavior tests were performed at a reservoir temperature of 36 °C.

### 5.3. Results of Liquid Polymer’s Stability

**Figure 6:**
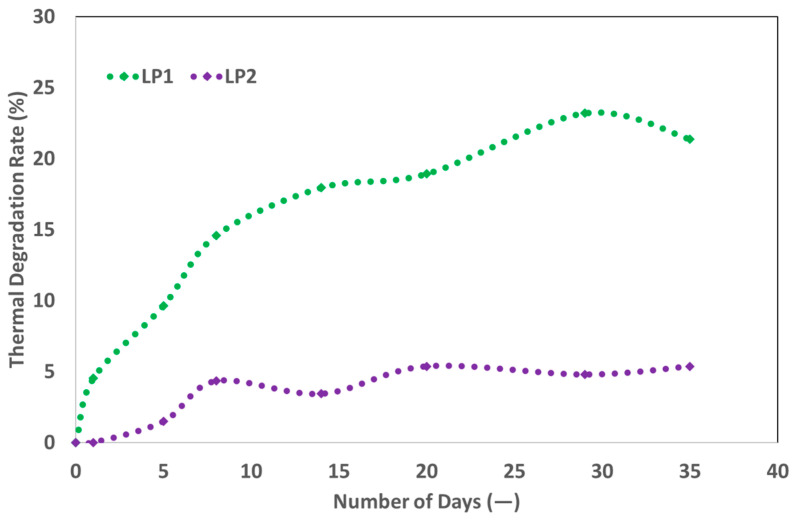
Thermal stability of liquid polymers LP1 and LP2 over a 35-day period at 36 °C. The graph shows viscosity degradation trends, indicating LP2’s superior thermal stability compared to LP1.

During the 35-day testing period, no obvious phase separation was seen in bottle tests for either liquid polymer at 36 °C. Nonetheless, as illustrated in Figure 6, both solutions exhibited a reduction in viscosity associated with thermal degradation. The viscosity reduction was higher for LP1 from vendor A compared to LP2 from vendor B. Consequently, the thermal stability tests determined that LP2 was the more stable product, owing to its extended residence time in reservoir during flooding at an expected flow rate of 1 ft/day under deep reservoir conditions.

### 5.4. Rock–Fluid Interactions

#### 5.4.1. Liquid Polymers

Targeting low- and medium-permeability zones, two types of core plugs (60 mD and 300 mD) with an average length of 7 cm and a diameter of 3.8 cm were used for single-phase core flood investigations. Liquid polymer injection encountered challenges, as the pressure in the 60 mD core plug consistently increased without achieving a steady state at injection rates between 1 ft/day (0.032 mL/min) and 15 ft/day (0.48 mL/min).

**Figure 7:**
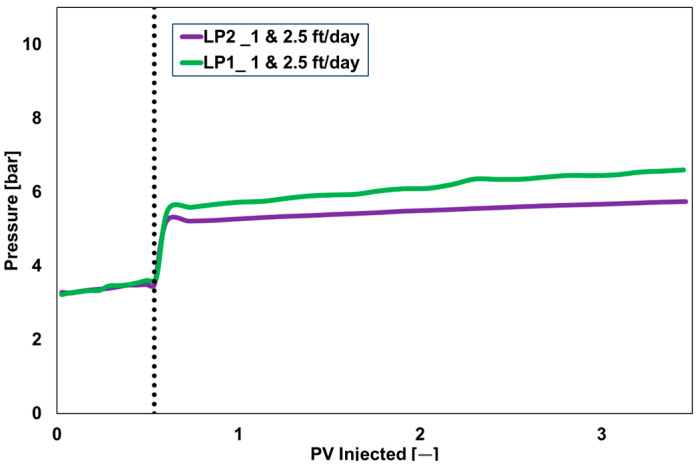
Pressure response during single-phase core flooding of liquid polymers LP1 and LP2 at injection velocities of 1 and 2.5 ft/day through 60 mD Berea sandstone core plugs. The figure highlights injectivity challenges and lack of pressure stabilization. Dashed line represents the change in injection velocity from 1ft/day to 2.5 ft/day.

**Figure 8:**
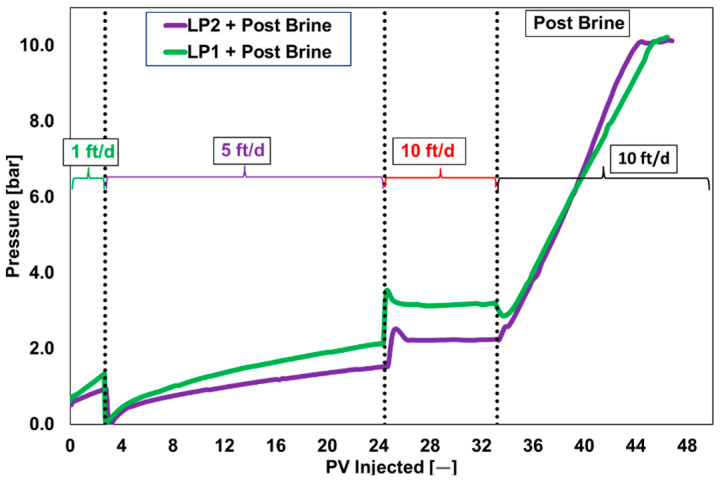
Single phase core flood for liquid polymers at various injection rates through 300 mD core plug. Dashed lines represent the change in injection velocities.

Figure 7 illustrates the outcomes of two liquid polymers introduced through the 60 mD core plug at injection velocities of 1 ft/day and 2.5 ft/day. Pressure stabilization was not accomplished, suggesting that the injection issue resulted from polymer filtering or blockage. A steady pressure difference was obtained by flooding powder polymer-based alkali polymers (PP1 and PP2) using low-permeability core plugs [33]. This suggests that the filtration of the base polymer was unlikely to be the cause of the injection issue. Consistent with the findings of Dwarakanath et al. [22] and Osterloh and Law [27], the emulsified phase (mineral oil and surfactant) appears to be the probable factor contributing to injection limitations. Consequently, we chose a core plug having higher permeability (300 mD) for further investigation. Figure 8 shows the LP injectivity outcomes in 300 mD core plug.

The 300 mD core plugs continued to have injection problems at all injection velocities except 10 ft/day. This implies that high injection rates that mimic near-wellbore conditions enhance the polymer’s injectability. Nonetheless, plugging concerns persisted at lower shear/injection rates, simulating the deep-in-reservoir condition. The primary concern was the rise in differential pressure for post-brine flood, as illustrated in Figure 8. At an injection velocity of 10 ft/day, the pressure increased until the injection pump approached its pressure limit. An increase in pressure was observed for products from both suppliers. Moreover, the suppliers validated similar pressure responses in their internal follow-up investigations.

One hypothesis argues that the chemical characteristics of both products are incompatible with the low permeability of the porous medium. Consequently, it is recommended to perform injection experiments utilizing high-permeability core plugs. Comparable injection problems have been reported with first-generation liquid polymers [22,27,34]. Core Flood studies indicated that the dispersed phase of the liquid polymer (an oil–water–polymer emulsion) was the primary factor leading to the injection issue [27]. A root cause investigation was performed by comparing the injection outcomes of the liquid polymer with those of a similar powdered polymer used as a base for the liquid polymer. In accordance with the findings in the literature [22,27], the injection of base powder polymers LP1 and LP2 into a 300 mD Berea sandstone core plug, as reported in our prior studies [35,36], demonstrated enhanced injectability and stable pressure behavior, suggesting that the powdered polymer was not a key factor contributing to injection limitations.

New-generation modified LP products have been developed, and the injection issue has been examined in prior research [22,27,34]. Nevertheless, considering the high permeability of the target reservoir (Captain Field), the studies employed highly permeable porous media. Our analysis of these qualifying LPs for Captain Field uncovered significant injection challenges when injecting LP into low-permeability reservoirs. The findings were communicated to the supplier, who agreed to develop new liquid polymers product that addresses the reservoir’s low permeability.

#### 5.4.2. Powder Polymers

It was decided to proceed with the polymer qualification process and consider powder polymers for the target reservoir (11 TH Sarmatian) because of injection problems noticed during LP testing. As indicated in Table 2 and Table 5, three more powder polymers were added to the screening process because powder polymer PP4 had been internally pre-approved for EOR polymer flooding based on prior testing. We achieved a target viscosity of 20 mPa·s at 20 °C and at a shear rate of 7.94 s^−1^ using the concentrations listed in Table 5. These concentrations also align with the supplier’s molecular weight information.

The first three powder polymer products (PP1, PP2, and PP3) were evaluated employing 300 mD core plugs for single-phase coreflood testing since PP4 had been internally pre-approved considering this permeability value.

**Figure 9:**
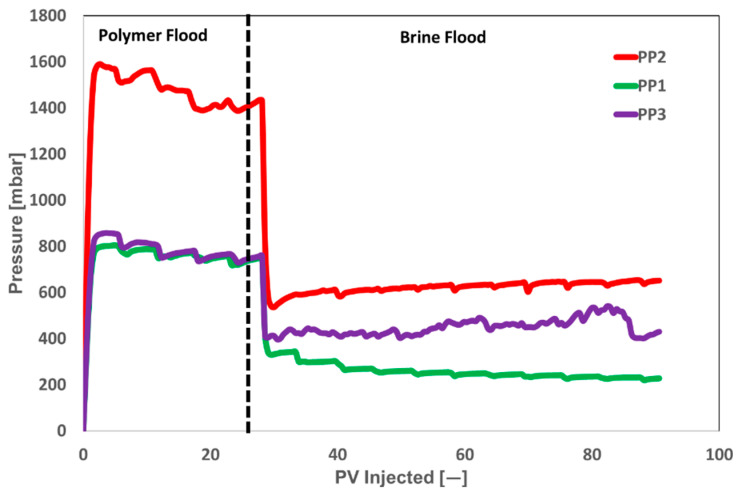
Single-phase core floods for three powder polymers at injection velocity of 10 ft/day in 300 mD core plugs. Dashed line represents the change in injected fluids.

Figure 9 shows single-phase polymer injectivity for a 300 mD core plug. The differential pressure is measured relative to the initial pressure of 0 millibar. The differential pressures for all three products were stable compared to the LP results, preventing injection issues. However, PP2 exhibited an enormous pressure drop (almost twice as much as PP1) even when the injection slug’s target viscosity was the same. It is still unclear how to accomplish a comparable field mobility ratio if the PP2 slug concentration is decreased to produce the same pressure drop in the core plug and increase EOR cost-effectiveness. The increased pressure drop for PP2 may be attributed to the product’s higher molecular weight, possibly resulting from a larger molecular weight distribution curve. Therefore, higher pressure is required to initiate slug injection into porous media.

**Figure 10:**
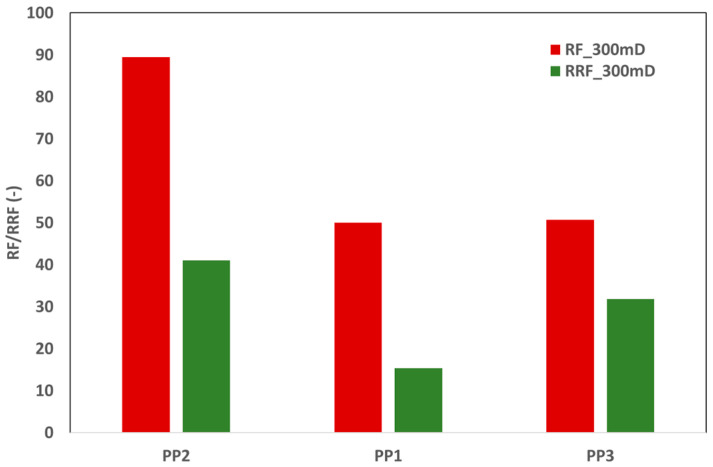
RF and RRF for three polymer products at injection velocity of 10 ft/day through 300 mD core plugs.

According to the pressure drop observed during polymer flooding, both products from vendor A (PP2 and PP3) demonstrated better injectivity. Subsequent brine flooding findings reveal that PP1 exhibits better performance due to its low pressure drop. Figure 9 illustrates that the higher pressure drops for PP2 and PP3 during brine flooding subsequent to polymer flooding signifies polymer adsorption, which affects core permeability. Figure 10 illustrates the resistance factor (RF) and residual resistance factor (RRF) obtained from pressure drop data during single-phase core flooding. Clearly, PP1 exhibits outstanding performance, characterized by both optimal injectivity (lower RF) and minimal polymer adsorption (lower RRF value).

**Figure 11:**
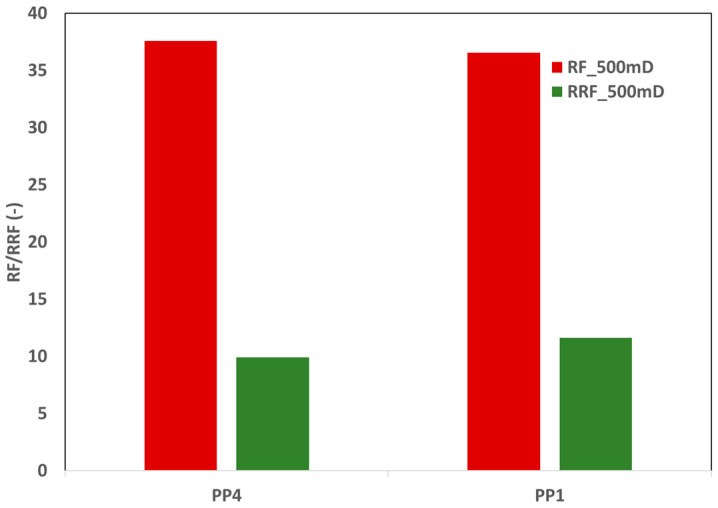
RF and RRF for two polymer products at injection velocity of 10 ft/day through 500 mD core plugs.

**Figure 12:**
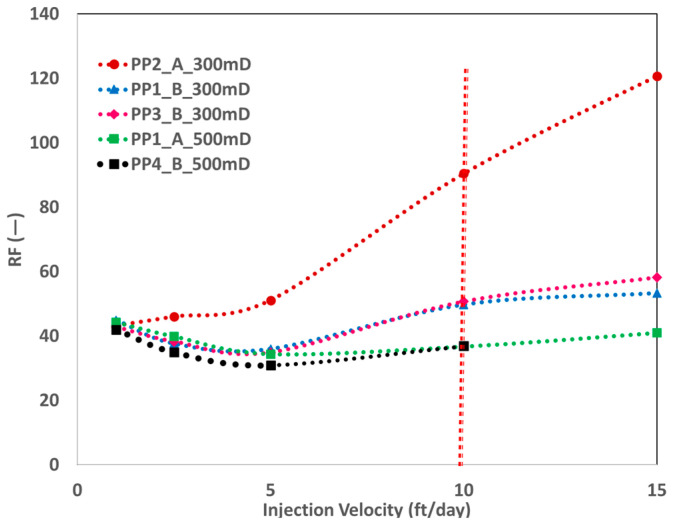
RF versus injection velocity for 300 mD and 500 mD—an indication for polymer viscoelasticity. Vertical dashed line represents the injection velocity of 10 ft/day.

Subsequently, single-phase core flood experiments were performed on 550 mD core plugs, reflecting the average permeability of the target reservoir. In the single-phase coreflood tests, the fluid injection rate was 10 ft/day. As previously stated, PP4 was an internally pre-approved product; nevertheless, PP1 was chosen because of its proven performance in single-phase core floods using a 300 mD core plug. Figure 11 illustrates that the RF and RRF performances of the two products are comparable. Moreover, the concentrations of the polymer slugs of both products were similar. Consequently, from the standpoint of polymer consumption and injectivity screening, both products exhibited similar findings. Figure 12 presents a further comparison of both products.

The resistance factor (RF) values of both products were similar at injection rates of 1 ft/day and 10 ft/day. Nevertheless, at these rates, the RF values for the 550 mD core plugs of PP1 were marginally superior to those of PP4 polymer. This result indicates a possibly increased amount of high-molecular-weight end traces in PP1. Figure 12 demonstrates that PP2 displays enhanced viscoelastic characteristics and does not exhibit shear-thinning behavior. The decrease in RF values with an increasing injection velocity signifies the polymer solution’s shear-thinning characteristics. The RF of PP2 exhibits a small increase from 1 ft/day to 5 ft/day. Nevertheless, after 5 ft/day, the slope of the RF curve grows steeper, signifying that viscoelasticity increases with the increase in the injection velocity (shear rate).

According to the molecular weight information provided by vendor A, PP3 possesses a slightly higher molecular weight than PP1. Figure 12 indicates that PP3 displayed slightly higher viscoelasticity at injection velocities above 10 ft/day. At lower injection rates, both products (PP1 and PP3) experienced identical behavior, specifically shear thinning followed by shear thickening. At an injection rate of 1 ft/day, consistent RF values were recorded for all solutions and both core plugs. This same behavior represents the Newtonian plateau of polymer slugs, as the viscosity of all injected fluids is similar.

#### 5.4.3. Mechanical Stability of Powder Polymers

**Figure 13:**
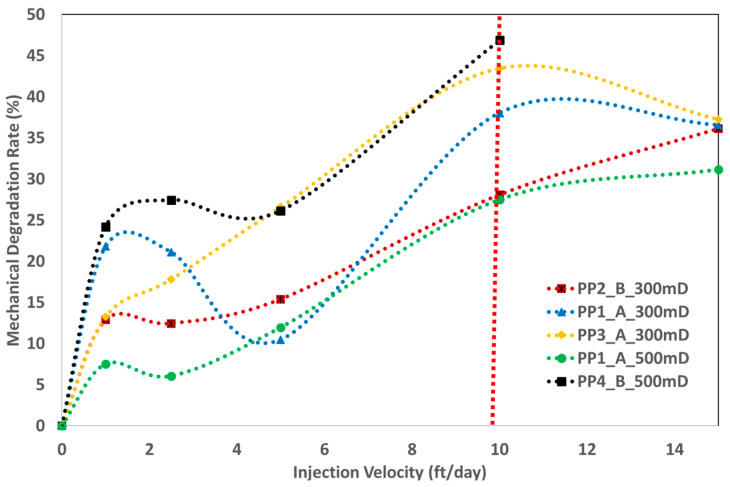
Mechanical stability of powder polymers as a function of injection velocity. Vertical dashed line represents the injection velocity of 10 ft/day.

The mechanical stability of the polymer slugs was evaluated using the mechanical degradation rate (MDR), as described in Equation (6). Core effluents were collected at each injection rate, and their viscosity was measured to determine the MDR. Figure 13 illustrates mechanical stability results. In the 300 mD core, PP2 demonstrated superior mechanical stability, as seen by the increased pressure drop during polymer injection in Figure 9 and the highest RF value in Figure 10. The higher RF value might have hindered injection because the PP2 solution showed a larger pressure drop upon pore penetration and maintained satisfactory viscosity.

Starting with the 550 mD core, PP4 demonstrated a substantial drop in viscosity within the porous medium compared to PP1. Consequently, the improved mobility of PP4 beyond the injection well appears questionable. PP1 demonstrated better mechanical stability compared to PP4. At higher injection velocities of 10–15 ft/day, the powdered polymer products demonstrated higher MDR. The breakdown of the polymer chains is expected to be caused by increased shear rates in the porous media.

### 5.5. Two-Phase Core Floods

**Figure 14:**
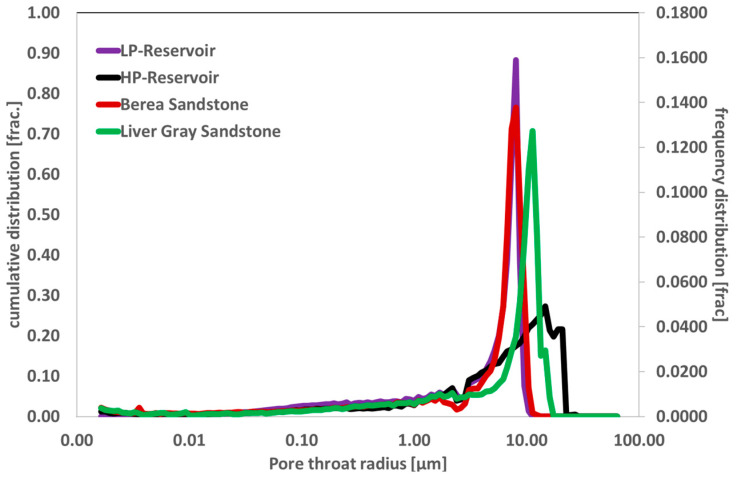
Pore size distribution correlation of reservoir plugs with outcrop plugs using MICP measurements.

The pore size distribution of outcrop cores and real reservoir core plugs was correlated using mercury injection capillary pressure (MICP) measurements, as illustrated in Figure 14.

Berea sandstone’s pore distribution and that of low-permeability (LP) reservoir cores showed a strong correlation. The Berea outcrop seems to be a promising porous media for two-phase core flooding since pore size distribution and pore throat diameter are critical factors for injectivity challenges and mobility control estimation. Additionally, the Liver Gray sandstone and high-permeability (HP) reservoir cores showed a notable offset response, which led to a poor correlation with the pore size distribution—a critical parameter for polymer injectivity. Consequently, Berea sandstone cores were chosen for two-phase core flooding.

**Figure 15:**
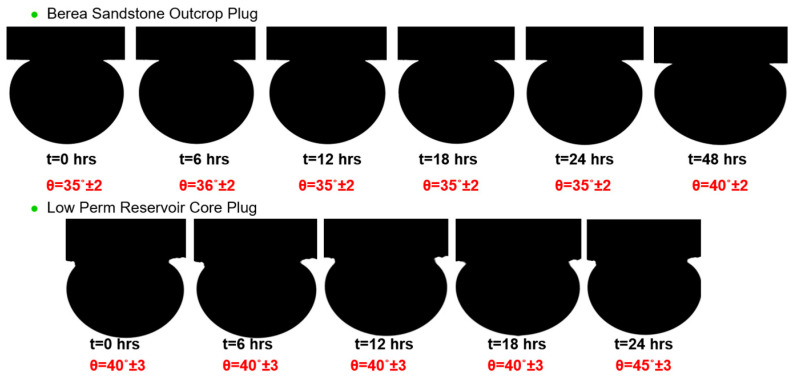
Contact angle measurements for low perm reservoir plug and Berea core plug.

To confirm the wettability of the reservoirs, further contact angle measurements were carried out on the LP reservoir cores and Berea sandstone. These measurements were carried out using a Dataphysics OCA 25L-PMC device (dataphysics 70794 Filderstadt, Germany). Oil droplets were injected from the bottom of a core plug using a needle and contact angle measurements were performed on cores saturated with brine in the aqueous phase. Both core plugs have strong water wettability, as shown by the contact angle measurements presented in Figure 15. As a result, it was decided not to perform the aging process for Berea core plugs before flooding with brine and polymers.

Two polymer products, PP1 and PP2, were used for two-phase corefloods. PP1 was chosen based on the results of single-phase corefloods, while PP2 was chosen for two reasons: the lower concentration required to achieve the target viscosity and similar polymer flood behavior at an injection rate of 1 ft/day during the single-phase coreflood. Two-phase corefloods at an injection rate of 1 ft/day were conducted to determine whether the pressure behavior of the polymer floods was consistent with the results of the single-phase corefloods.

**Figure 16:**
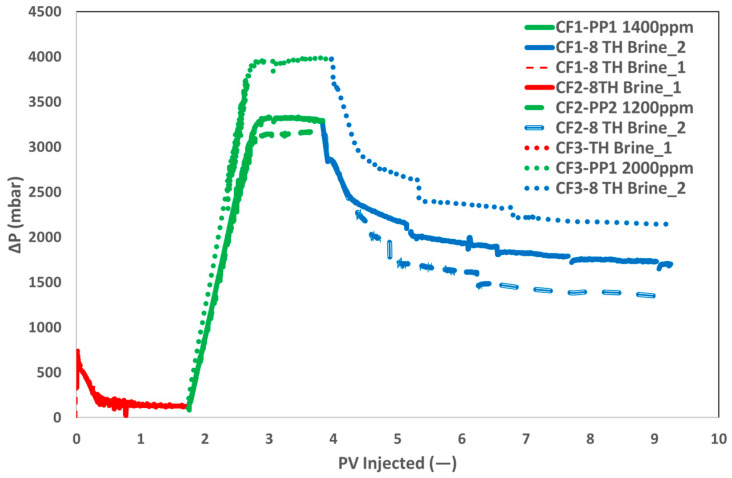
Pressure differential profiles during two-phase core flooding experiments using PP1 and PP2 polymers. The graph compares pressure behavior across brine flood (red), polymer flood (green), and post-brine flood (blue) stages, illustrating injectivity and viscoelastic effects at 1 ft/day injection velocity.

Figure 16 shows the pressure drop during the two-phase corefloods. Similar pressure behavior was observed during the brine flood in all three corefloods. Due to the similar polymer viscosities, comparable pressure drops were observed during the polymer floods for both polymer products (CF1 and CF2). Therefore, at an injection rate of 1 ft/day, the pressure behavior of both products was identical. This suggests that the higher-pressure differential observed in the single-phase coreflood at an injection rate of 10 ft/day is due to the viscoelastic properties of PP2. This further demonstrates that at higher injection rates, PP2 induces higher pressure differentials near the wellbore, while at lower injection rates, the two polymer products behave similarly deeper in the reservoir. This conclusion is based solely on the comparisons conducted under laboratory conditions in this paper. It is important to note that the behavior observed in a controlled environment is difficult to generalize to field conditions.

A third core flood was conducted with a higher PP1 concentration to compare the impact of increased viscosity. Although the slug’s viscosity was almost twice as high as in the baseline scenario (37 mPa·s vs. 20 mPa·s), Figure 16 illustrates that the pressure drop did not double. This pressure response results from the polymer slug’s non-Newtonian nature and dependence on the in situ shear rate, which causes the polymer chains to mechanically break down. It is obvious to see how the pressure drop decreased during the brine flush. At the end of the post-brine flood, PP1 showed a marginally higher pressure drop than PP2 in comparison to the single-phase core flood. As anticipated, CF3’s higher polymer concentration led to a higher pressure drop at the post-brine flood’s conclusion as compared to CF1.

**Figure 17:**
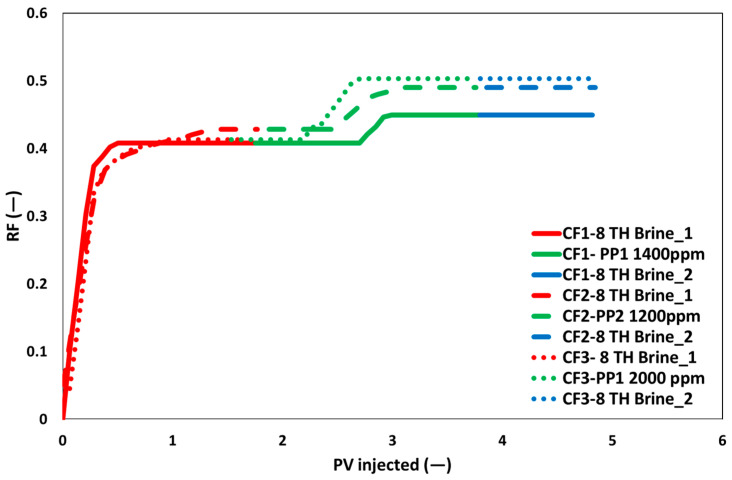
Oil recovery response for two-phase core flood for the brine flood (red color) polymer flood (green color) and post brine flood (blue color).

The recovery curves for the three core floods are displayed in Figure 17. The routine core analysis of the core plugs and the additional recovery contributions of the polymer slugs are also reported in Table 6. Initial oil saturation and rock characteristics were almost the same because of core plug homogeneity. Oil recovery factors (ORF) from brine flooding were comparable, with 2% variation. The difference is within the measurement error of core flood repeatability. The next polymer flooding yielded comparable additional ORF values of roughly 5% for both polymer slugs (CF1 and CF2) having identical viscosities. Due to one-dimensional flow and the lack of any fluid–fluid interaction, the lower ORF for the 300–400 mD core plug with polymer flooding is in alignment with our previous findings [36,37,38,39].

Remarkably, there was only an increase in ORF of 8% when the slug viscosity was increased (doubled) for CF3. The low additional ORF value of 3% is related to the insignificant increase in pressure drops, as shown in Figure 16. Brine flooding did not contribute to further ORF in any of the three core floods. Consequently, it may be concluded that an increase in polymer viscosity does not lead to a proportional increase in additional oil recovery, attributed to the non-Newtonian characteristics of the polymer slugs. Consequently, an economic analysis must be performed to determine the necessary increased polymer concentration in relation to the additional oil recovery obtained.

**Figure 18:**
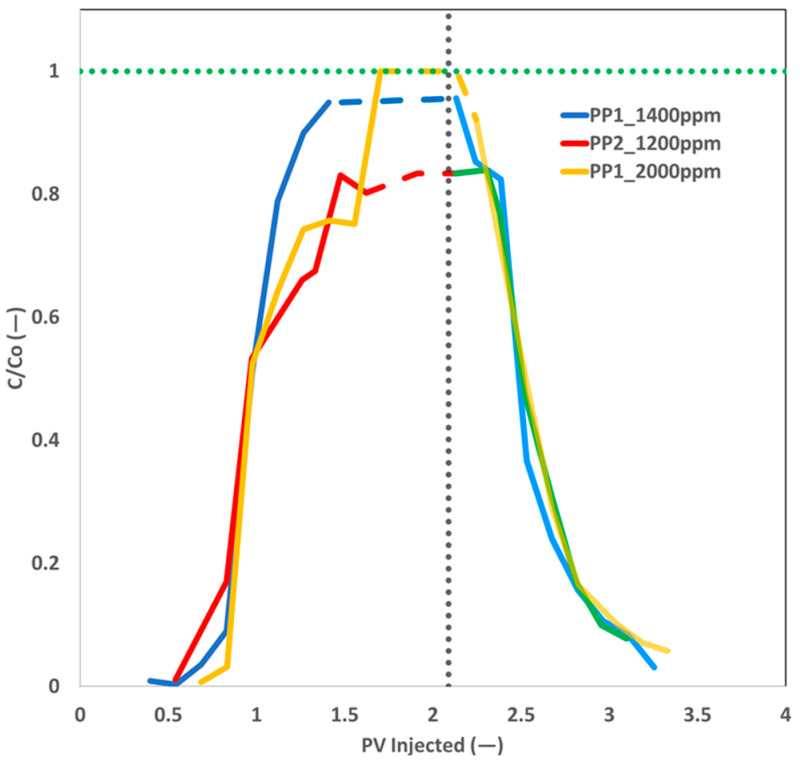
Polymer analytics for polymer flooding and post brine flooding. Vertical dashed line represents the change in injection fluids.

The polymer analytics results for the three core floods are displayed in Figure 18. The effluent concentration for the PP1 polymer plug was comparable to that of the injected plug. On the other hand, the PP2 polymer slug’s effluent concentration was slightly lower than that of the polymer slug. A possible reason for the distinction is that the PP2 product exhibits higher adsorption/entrapment capabilities than the PP1 product. Additional testing is required to validate this either adsorption or entrapment concept. The persistent decline in polymer concentration across the three core floods indicates polymer washout from the core plugs. Nevertheless, even after an injection exceeding 1 PV, the polymer concentration did not drop to zero.

## 6. Discussion

### 6.1. Cost Comparison

Cost comparison was performed by estimating polymer consumption rates, unit costs, and infrastructure requirements. A study by Hezekiah-Braye et al. [40] presents a detailed and professional economic analysis of polymer flooding materials; however, we present a simplified cost comparison based on the major cost-driving and critical factors. Liquid polymers, while easier to deploy, incur higher operational costs due to lower active content and higher price per kg. Powder polymers, though requiring more space and initial investment, offer better long-term cost efficiency. Over a 3-year period, LP is more economical, but over a 10-year horizon, powder polymers become more cost-effective. These estimates are based on normalized cost units derived from vendor data and internal logistics modeling.

The cost comparison uses LP as the baseline (100 units). Over 3 years, PP Option 1 costs 130 units (30% more), and PP Option 2 costs 50 units (50% less). Over 10 years, LP costs 150 units (50% more than both PP options, which are 100 units each). These normalized values reflect total cost impact including CAPEX, OPEX, logistics, and operational feasibility. These units are not in euros or millions, but rather normalized cost indices for comparison purposes. Instead, we use relative cost comparisons and percentage differences to illustrate the economic feasibility of each polymer flooding option.

- Liquid Polymer (LP) is used as the baseline (100 units).
- Powder Polymer Option 1 is said to cost 30% more than LP over 3 years → interpreted as 130 units.
- Powder Polymer Option 2 is 50% cheaper than LP over 3 years → interpreted as 50 units.
- Over 10 years, LP is 50% more expensive than both powder options → LP = 150 units, PP1 & PP2 = 100 units.

#### 6.1.1. Cost Estimation Logic

Polymer Consumption Assumptions

- Liquid Polymer (LP): 240 kg/day (due to 50% active content).
- Powder Polymer (PP): 120 kg/day.

Relative Cost per kg

- LP is 60% more expensive per kg than PP.
- LP requires double the quantity to achieve the same active polymer content.

#### 6.1.2. Scenario-Based Cost Comparison

Table 7 compares three polymer flooding scenarios—Liquid Polymer (LP), Powder Polymer Option 1, and Option 2—based on cost, space requirements, and operational complexity. It highlights trade-offs between short-term and long-term feasibility to support decision-making for field deployment.

#### 6.1.3. Relative Cost Outcomes

**Figure 19:**
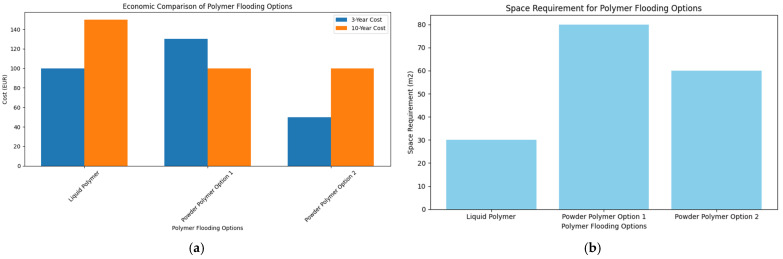
(**a**) Cost comparison of polymer flooding options over 3 and 10 years; (**b**) Space requirements for different polymer flooding setups.

Table 8 and Figure 19 present a comparative analysis of polymer flooding costs over 3-year and 10-year horizons. The values are normalized cost indices (not actual EUR figures) and reflect total cost impact, including capital expenditure (CAPEX), operational expenditure (OPEX), logistics, and operational feasibility.

For the 3-year horizon, liquid polymer (LP) is used as the baseline at 100 units. Powder Polymer Option 1 incurs 30% higher cost (130 units), while Option 2 is 50% cheaper (50 units). Over a 10-year horizon, LP becomes 50% more expensive than both powder options, with LP at 150 units and both powder options at 100 units. These trends suggest that LP may be more economical for short-term projects due to lower initial CAPEX, but powder polymers offer better cost efficiency for long-term operations.

Table 8 also includes space requirements and operational complexity, which are critical for field deployment. LP requires only 30 m^2^ and has low operational complexity, making it suitable for space-constrained sites. In contrast, Powder Polymer Option 1 requires 80 m^2^ and is operationally complex, while Option 2 requires 60 m^2^ with medium complexity. Figure 19a visualizes the cost comparison across time horizons, and Figure 19b illustrates the spatial footprint of each setup, aiding in strategic decision-making for pilot implementation.

### 6.2. Decision Matrix

Table 9 presents the decision matrix summarizing the performance of polymer products based on single-phase and two-phase core flooding experiments. Liquid polymers were excluded due to injectivity challenges, while powdered polymers demonstrated acceptable injectivity and were further evaluated. Among them, PP1 showed superior performance in terms of mechanical stability, injectivity at near-wellbore conditions (low RF), and minimal polymer retention (low RRF). Although PP1 required a slightly higher dosage to achieve the target viscosity, its overall performance supports its selection for pilot implementation. Product cost remains a consideration in the final selection process.

Table 9 is structured to compare polymer products across key performance indicators including injectivity (RF), retention (RRF), mechanical stability, and oil recovery factor (ORF). The matrix uses core plug data from 300 mD and 550 mD samples to reflect near-wellbore and average reservoir conditions. Each polymer is evaluated based on its ability to maintain viscosity, minimize pressure drop, and enhance oil recovery. This comparative framework aids in selecting the most suitable polymer for pilot testing under space-constrained reservoir conditions.

Based on the completed technology qualification process for the reservoir conditions of the 11 TH Sarmatian reservoir in the Hochleiten field, PP1 was recommended for initiation as a pilot project. Given the lower polymer concentration required to achieve the target viscosity and the resulting economic benefits, PP2 could also be tested as a pilot project at a later stage. Because PP1 and PP2 contributed similar additional oil production and given the deep reservoir conditions (1 ft/day), similar polymer flooding pressure responses were observed at lower injection rates.

While the decision matrix provides a structured comparison of polymer performance, several limitations must be acknowledged. The results are based on laboratory-scale core flood experiments, which may not fully replicate field conditions due to scale effects and operational variability. Additionally, the representativeness of the core plugs—especially outcrop samples—may not capture the full heterogeneity of the reservoir. Surfactant interactions, particularly in liquid polymer formulations, were not extensively evaluated and could influence injectivity and retention in actual field applications. These factors should be considered when extrapolating laboratory findings to field-scale implementation.

## 7. Conclusions

We have presented a comprehensive evaluation of liquid and powder polymers for enhanced oil recovery (EOR) in a low-permeability, space-constrained Austrian reservoir. The findings highlight significant differences in injectivity, stability, recovery performance, and economic feasibility between the polymer types.

- Rheological and Thermal Stability: All polymers achieved the target viscosity of 20 mPa·s at 20 °C and a shear rate of 7.94 s^−1^. LP2 showed better thermal stability than LP1, with lower viscosity degradation over 35 days at 36 °C. Powder polymers maintained viscosity across temperature ranges, with PP3 and PP4 showing slightly higher values (21 mPa·s at 20 °C).
- Injectivity and Coreflood Performance: Liquid polymers LP1 and LP2 exhibited injectivity challenges in 60 mD and 300 mD Berea sandstone core plugs, with pressure stabilization not achieved at injection rates of 1–2.5 ft/day. In contrast, powder polymers demonstrated stable injectivity, with PP1 achieving consistent pressure behavior at 10 ft/day and exhibiting low resistance factor (RF = 50) and residual resistance factor (RRF = 15) in 300 mD cores. PP2 showed higher RF (89) and RRF (41), indicating greater retention and pressure drop.
- PP1 and PP2 exhibited similar pressure behavior at low injection rates (1 ft/day) due to comparable viscosities; however, PP2 showed significantly higher pressure differentials at higher rates, likely due to its viscoelastic properties. These findings, derived from controlled laboratory conditions, may not directly translate to field performance.
- Recovery Efficiency: Two-phase core floods using PP1 and PP2 at 1 ft/day yielded incremental oil recovery factors (ORF) of 5% and 6%, respectively. A third test with a higher PP1 concentration (2000 ppm) resulted in an ORF of 8%, demonstrating that increased viscosity does not proportionally enhance recovery due to non-Newtonian flow behavior.
- Economic Feasibility: Over a 3-year horizon, LP is more economical (100 units baseline), while PP Option 1 costs 130 units and Option 2 only 50 units. Over a 10-year horizon, LP becomes 50% more expensive (150 units) compared to both powder options (100 units). LP systems require only 30 m^2^ and offer low operational complexity, whereas PP Option 1 requires 80 m^2^ and high complexity, and Option 2 requires 60 m^2^ with moderate complexity.
- Polymer Selection: PP1 was selected for pilot implementation due to its superior injectivity, mechanical stability, and recovery performance. PP2 is a viable alternative, offering similar recovery at lower concentrations but with higher retention. Liquid polymers were disqualified due to injectivity limitations and emulsion-related filtration issues.
- Limitations and Future Work: Laboratory-scale results may not fully replicate field conditions due to scale effects and reservoir heterogeneity. Core plug representativeness and surfactant interactions, especially in LP formulations, require further investigation. Field-scale validation and long-term performance monitoring are recommended to confirm laboratory findings and optimize polymer flooding strategies.

In conclusion, although liquid polymers pose injection issues in low-permeability reservoirs, powdered polymers, especially PP1, exhibited improved efficiency and are recommended for this pilot project.

## Figures and Tables

**Table 1:** Brine composition used for this study.

Salt	8 TH WTP (g/L)
NaCl	22.48
KCl	0.16
MgCl_2_ × 6H_2_O	0.63
CaCl_2_ × 2H_2_O	0.94
NaHCO_3_	-
TDS	24.21

**Table 2:** Liquid and powder polymer used for this study.

Polymer	Type	Vendor	Product Proposal by	Core Flood
LP1	Liquid Polymers	VendorA	Vendor	Single Phase
LP2		VendorB	Vendor	Single Phase
PP1	Powder Polymers	VendorA	Vendor	Single Phase + Two Phase
PP2		VendorB	Vendor	Single Phase + Two Phase
PP3		VendorA	Internal	Single Phase
PP4		VendorB	Internal	Single Phase

**Table 3:** Polymer product concentrations and core plugs selected for this study.

Polymer	Vendor	Concentration (ppm)	Core Plug (mD)
LP1	A	1200	300 & 60
LP2	B	2250	300 & 60
PP1	A	1400	300 & 550
PP2	B	1200	300
PP3	A	1300	300
PP4	B	1400	550

**Table 4:** Selected polymers used for two-phase core flood.

Exp. #	Polymer Concentration	Slug Type	Core Type	Comment
1	1400 ppm PolymerA	VendorA	Berea	Base case
2	1200 ppm PolymerB	VendorB		Base case
3	2000 ppm PolymerA	VendorA		High concentration case

**Table 5:** Concentration and temperature scan for both polymer products.

Polymers	Vendor	Concentration (ppm)	Viscosity (mPa·s)		
			20 °C	25 °C	36 °C
LP1	A	1200	20	19	16
LP2	B	2250	20	18	16
PP1	A	1400	19	18	16
PP2	B	1200	20	19	16
PP3	A	1300	21	20	17
PP4	B	1400	21	21	17

**Table 6:** RCA and oil recovery factor summary for two phase core floods.

ID	Porosity	PV	Perm. (Brine)	Kro * @ Swi *	Krw * @ Sros *	Slug	Soi *	Brine ORF	Polymer ORF	Brine ORF
	(%)	mL	mD	(−)		(−)	(%)	(−)		
CF1	20.4	69	384	0.81	0.047	1400 ppm PP1	70	41	5	0
CF2	20.4	69	395	0.81	0.048	1200 ppm PP2	71	43	6	0
CF3	20.3	69	411	0.79	0.050	2000 ppm PP1	70	42	8	0

* Where Soi = Initial oil saturation, Swi = Initial brine saturation, kro = Relative perm. of oil, Krw = Relative perm. of brine and Sros = Remaining oil saturation.

**Table 7:** General Description for the Scenario-Based Cost Comparison.

Scenario 1 (LP):	Scenario 2 (PP Option 1):	Scenario 3 (PP Option 2):
Lower CAPEX due to compact, containerized unit. Higher OPEX due to higher polymer cost and consumption. Can be placed directly at the wellhead (no pipeline cost).	High CAPEX due to large mixing tanks and infrastructure. Lower OPEX due to cheaper polymer and lower consumption. Requires pipeline (1.5 km) and more space.	Moderate CAPEX (offsite preparation). Risk of degradation during transport. Similar OPEX to Scenario 2.

**Table 8:** Scenario-Based Cost Comparison.

Scenario	3-Year Cost (EUR)	10-Year Cost (EUR)	Space Requirement (m^2^)	Operational Complexity
Liquid Polymer	100	150	30	Low
Powder Polymer Option 1	130	100	80	High
Powder Polymer Option 2	50	100	60	Medium

**Table 9:** Decision matrix for polymer product selection.

Polymer Product	LP1	LP2	PP2	PP3	PP1	PP4
Target Concentration	1200 ppm	2250 ppm	1200 ppm	1300 ppm	1400 ppm	1400 ppm
Near Wellbore Injectivity (RF)	No P stability	No P stability	89 *	51 *	50 * & 37 **	38 **
Deep in reservoir (RF)	No P stability	No P stability	43 *	43 *	45 * & 44 **	42 **
Near Wellbore Stability of Polymer (Vis. Loss %)	Filtration	Filtration	28 *	43 *	38 * & 28 **	47 **
Deep in Reservoir Stability of Polymer (Vis. Loss %)	Filtration	Filtration	13 *	13 *	22 * & 8 **	24 **
RRF/Retention	NA	NA	41 *	32 *	15 * & 12 **	10 **

* 300 mD core plugs. ** 550 mD core plugs.

## Data Availability

The original contributions presented in this study are included in the article. Further inquiries can be directed to the corresponding author.

## Abbreviations

CAPEX	Capital Expenditure
cEOR	Chemical Enhanced Oil Recovery
EOR	Enhanced Oil Recovery
HL	Hochleiten
HPAM	Hydrolyzed Polyacrylamide
IFT	Interfacial Tension
LP	Liquid Polymer
MDR	Mechanical Degradation Rate
MICP	Mercury Injection Capillary Pressure
ORF	Oil Recovery Factor
RF	Resistance Factor
RRF	Residual Resistance Factor
TAN	Total Acid Number
TDS	Total Dissolved Solids
WTP	Water Treatment Plant
